# A Multidirectional Perspective for Novel Functional Products: *In vitro* Pharmacological Activities and *In silico* Studies on *Ononis natrix* subsp. *hispanica*

**DOI:** 10.3389/fphar.2017.00600

**Published:** 2017-09-01

**Authors:** Serife Yerlikaya, Gokhan Zengin, Adriano Mollica, Mehmet C. Baloglu, Yasemin Celik Altunoglu, Abdurrahman Aktumsek

**Affiliations:** ^1^Department of Genetics and Bioengineering, Faculty of Engineering and Architecture, Kastamonu University Kastamonu, Turkey; ^2^Department of Biology, Science Faculty, Selcuk University Konya, Turkey; ^3^Department of Pharmacy University “G. d'Annunzio” of Chieti-Pescara Chieti, Italy

**Keywords:** *Ononis*, bioactive compounds, natural agents, pharmaceuticals, molecular docking

## Abstract

The genus *Ononis* has important value as traditional drugs and foods. In the present work, we aimed to assess the chemical profiles and biological effects of *Ononis natrix* subsp. *hispanica* extracts (ethyl acetate, methanol, and water). For chemical profile, total and individual phenolic components were detected. For biological effects, antioxidant (DPPH, ABTS, CUPRAC, FRAP, phosphomolybdenum, and metal chelating assays), enzyme inhibitory (against cholinesterase, tyrosinase, α-amylase and α-glucosidase), antimicrobial, DNA protection and cytotoxic abilities were tested. The predominant phenolics were apigenin, luteolin, and quercetin in the tested extracts. Generally, the ethyl acetate and methanol extracts were noted as the most active in the antioxidant and enzyme inhibitory assays. Water extract with different concentrations indicated high level of DNA protection activity. Methanol and ethyl acetate extracts showed antibacterial effect against to *Staphylococcus aureus* and *Staphylococcus epidermidis* strains. The cytotoxic effects of *O. natrix* subsp. *hispanica* extracts on the survival of HeLa and PC3 cells were determined by MTT cell viability assay. Water and methanol extracts caused initiation of apoptosis for PC3 cell line. Furthermore, molecular docking was performed to better understand interactions between dominant phenolic compounds and selected enzymes. Our results clearly indicate that *O. natrix* subsp. *hispanica* could be considered a potential candidate for designing novel pharmaceuticals, cosmeceuticals and nutraceuticals.

## Introduction

In the twenty first century, the usage of plant or plant products are undergoing a revolution as sources of prominent (functional and bioactive) compounds. For example, artemisinin was awarded as an anti-malarial compound in Nobel Prize for Medicine 2015 (Omura and Campbell, 2015). From this point, to better combat the lifestyle, communicable or infectious diseases, many phytochemicals have been suggested as antioxidant, antimicrobial, anticancer and anti-mutagenic agents (Fernandes et al., 2017; Losada-Barreiro and Bravo-Díaz, 2017; Meeran et al., 2017; Yin et al., 2017). These facts provide encouragement for designing further studies on the plants. Within these perspectives, the term of functional products has emerged and it is defined as any products which provide health benefits including treatment of afore-mentioned diseases. In this framework, uninvestigated wild plants could be considered as a springboard for designing novel functional products with low toxicity.

The genus *Ononis* (Fabaceae) is represented by 75 species in the world especially Europe and Central Asia (Wollenweber et al., 2003). The genus comprises of 23 taxa in Tukey and known as “semisk” and “kayışkıran” in Turkish name (Baytop, 1999; Sohretoglu, 2007; Mükemre et al., 2015). In the literature, several studies were reported that member of the genus *Ononis* exhibited significant biological activities, such as antimicrobial effect (Mhamdi et al., 2015; Sayari et al., 2016), antioxidant (Mhamdi et al., 2015; Mezrag et al., 2017), wound healing (Süntar et al., 2011), cytotoxic (Kuete et al., 2013; Ghribi et al., 2016), and analgesic (Yõlmaz et al., 2006). In addition, the decoctions from *Ononis* species is used for treating urinary problems, skin disorders as well as gout (Baytop, 1999; Erdemgil et al., 2002; Liebezeit, 2008). With this in mind, *Ononis* species have been sold as herbal tea on the market as single or combined with other diuretic plants (Gampe et al., 2016). Several publications have appeared in recent years documenting on biological activities of some *Ononis* species along with their phytochemicals profiles (Daruházi et al., 2008; Al-Rehaily et al., 2014; Ghribi et al., 2015; Yousaf et al., 2015; Gampe et al., 2016; Casiglia et al., 2017; Mezrag et al., 2017). However, to the best of our knowledge, there is no report on the chemical profile and biological ability of *Ononis natrix* subsp. *hispanica*. Thus, the present study aims to provide more details about chemical and biological properties of *O. natrix* subsp. *hispanica*. The observed findings could be provide new insights for *O. natrix* subsp. *hispanica*.

## Materials and methods

### Plant material and preparation of the extracts

*Ononis natrix* subsp. *hispanica* was collected from Antalya-Turkey during of flowering season (June 2015). Taxonomic identification of the plant material was confirmed by the senior taxonomist Dr. Murad Aydin Sanda, from the Department of Biology, Selcuk University. The voucher specimen has been deposited at the Herbarium of the Department of Biology, Selcuk University, Konya-Turkey.

To obtain ethyl acetate and methanol extracts, the air-dried aerial parts (10 g) were macerated with 200 mL of these solvents at room temperature (25° ± 1°C) for 24 h. The extracts were concentrated under vacuum at 40°C by using a rotary evaporator. To obtain water extract, the powdered samples were boiled with 250 mL of distilled water for 30 min. The aqueous extract was filtered, lyophilized (−80°C, 48 h), and all extracts stored at + 4°C in the dark until use.

### Total phenolics, flavonoids, and phenolic composition

The total phenolics content was determined by Folin-Ciocalteu method (Slinkard and Singleton, 1977) with slight modification and expressed as gallic acid equivalents (GAE/g extract), while total flavonoids content was determined using AlCl_3_ method (Zengin et al., 2014) with slight modification and expressed as rutin equivalents (RE/g extract).

Phenolic compounds were evaluated by RP-HPLC (Shimadzu Scientific Instruments, Tokyo, Japan). Detection and quantification were carried out with a LC-10ADvp pump, a Diode Array Detector, a CTO-10Avp column heater, SCL-10Avp system controller, DGU-14A degasser and SIL-10ADvp auto sampler (Shimadzu Scientific Instruments, Columbia, MD). Separations were conducted at 30°C on Agilent® Eclipse XDB C-18 reversed-phase column (250 × 4.6 mm length, 5 μm particle size). Phenolic compositions of the extracts were determined by a modified method of Zengin et al. (2014). Gallic acid, protocatechuic acid, (+)-catechin, p-hydroxybenzoic acid, chlorogenic acid, caffeic acid, (−)-epicatechin, syringic acid, vanillin, *p-*coumaric acid, ferulic acid, sinapic acid, benzoic acid, *o-*coumaric acid, rutin, hesperidin, rosmarinic acid, eriodictyol, *trans-*cinnamic acid, quercetin, luteolin, kaempferol, and apigenin were used as standards. Identification and quantitative analysis were done by comparison with standards. The amount of each phenolic compound was expressed as microgram per gram of extract using external calibration curves, which were obtained for each phenolic standard.

### Antioxidant and enzyme inhibitory assays

Antioxidant [DPPH, ABTS radical scavenging, reducing power (CUPRAC and FRAP), phosphomolybdenum and metal chelating (ferrozine method) and enzyme inhibitory assays (cholinesterase (Elmann's method), tyrosinase (dopachrome method), α-amylase (iodine/potassium iodide method) and α-glucosidase (chromogenic PNPG method)] were performed according to our previous researches (Zengin et al., 2014; Zengin, 2016). Antioxidant capacities were expressed as equivalents of trolox and EDTA (for metal chelating). In addition, the enzyme inhibitory activities of the extracts were evaluated as equivalents of standard inhibitors per gram of the plant extract (galantamine for AChE and BChE, kojic acid for tyrosinase, and acarbose for α-amylase and α-glucosidase inhibition assays).

### Molecular modelling

#### Receptors preparation

The crystalline structure of the selected enzymes together with their inhibitors have been downloaded from the Protein Databank RCSB PDB (Berman et al., 2000): acetylcholinesterase (pdb:4X3C) (Pesaresi and Lamba, in press) in complex with tacrine-nicotinamide hybrid inhibitor, butyrilcholinesterase (pdb:4BDS) (Nachon et al., 2013) in complex with tacrine, amylase (pdb:1VAH) (Zhuo et al., 2004) in complex with r-nitrophenyl-α-D-maltoside, glucosidase (pdb:3AXI) (Yamamoto et al., 2011) in complex with maltose and tyrosinase (pdb:2Y9X) (Ismaya et al., 2011) in complex with tropolone. The raw crystal structures were prepared for the docking experiments as previously reported (Mocan et al., 2016; Uysal et al., 2016). Non-catalytic water molecules, inhibitors and all the other molecules present in the pdb files were removed by using UCSF Chimera (DeLano, 2002) and the proteins alone were neutralized at pH 7.4 by PropKa implemented in Maestro 10.2 suite (Maestro, 2015). Seleno-cysteines and seleno-methionines, if present, were converted to cysteines and methionines, respectively. All the missing fragments and other errors present in the crystal structures were automatically solved by the Wizard Protein Preparation implemented in Maestro 10.2 suite (Maestro, 2015).

#### Ligands preparation

**Figure 1:**
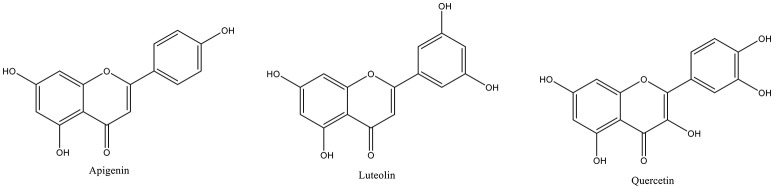
Chemical structure of apigenin, luteolin and quercetin.

(+)-Epicatechin, apigenin, luteolin, quercetin and rosmarinic acid were selected as representative compounds to carry out molecular docking study, as these compounds were present in abundance in the herbs extracts. The chemical structure the selected compounds are reported in Figure 1. The three dimensional structures have been downloaded from Zinc databases (Irwin et al., 2012) and used for molecular modeling experiments after preparation. The ligands were prepared by the LigPrep tool embedded in Maestro 10.2, neutralized at pH 7.4 by Ionizer and minimized by OPLS3 force field (Shelley et al., 2007).

### Molecular docking

Dockings of the representative substances have been performed for each selected enzyme employed for the *in vitro* enzymatic inhibition tests in this work. Glide embedded in the maestro suite 10.2 has been employed for the docking calculations by using the “eXtra Precision” scoring function for all the enzymes and the mm-GBSA binding energy has been calculated by the use of Prime embedded in maestro 10.2 (Table 1; Jones et al., 1997). In all cases, the binding pocket was determined automatically by centering the grid on the crystallographic inhibitor, extended in a radius of 10 Angstroms from the center. The best pose for each compound docked to the selected enzymes was the best ranked among the 10,000 generated.

**Table 1:** Binding energy and glide XP docking scores.

**Compounds**	AChE^*^		BChE^*^		α-amylase^*^		α-glucosidase^*^		Tyrosinase^*^	
	**XP**	**ΔG**	**XP**	**ΔG**	**XP**	**ΔG**	**XP**	**ΔG**	**XP**	**ΔG**
Apigenin	−9.6	−60.6	−9.4	−52.5	−5.98	−35.2	−5.7	−17.2	−5.7	−26.1
Benzoic A.	−4.0	−14.2	−4.9	−22.3	−3.88	−0.03	−2.3	+71.8	−5.1	+41.7
(−)-Epicat.	−9.3	−41.5	−7.3	−41.0	−7.03	−38.1	−6.9	−21.8	−5.5	−20.9
Luteolin	−9.6	−52.9	−8.0	−53.3	−6.54	−54.0	−6.7	−30.3	−5.1	−29.3
Quercetin	−10.3	−62.9	−7.9	−43.5	−8.79	−52.9	−6.9	−31.2	−5.4	−28.3
Rosmarinic A.	−11.3	−64.5	−10.9	−42.8	−8.26	−46.8	−6.5	−22.6	−6.3	−12.2

* *ΔG values (Binding energy) are expressed in Kcal/mol*.

### DNA protection

DNA protection activity of the studied extracts was analyzed using pUC19 plasmid DNA (pDNA). Plasmid isolation was performed by Thermo Scientific Genejet Plasmid Miniprep Kit. The reaction mixture contained 5 μL of Fenton's reagent (30 mM H_2_O_2_, 50 mM ascorbic acid, and 80 mM FeCI_3_), 5 μL of these extracts at two different concentrations (5 and 10 mg/mL) and 3 μL of pDNA (300 μg/μL). Final volume of reaction mixture was brought up to 20 μL using double-distilled water. Positive control was composed of 12 μL of distilled water, 5 μL of Fenton's reagent and 3 μL of pDNA. Negative control involved only 17 μL of distilled water and 3 μL of pDNA. Samples were incubated for 30 min at 37°C and 4 μL loading dye (Thermo Scientific, USA) was added to the all mixtures. The DNA mixtures were run on 0.8% agarose gel and then visualized under ultraviolet light cabin. Biological replication of test was carried out at three times and band density was determined by the gel image analysis software (Quantum, Vision-Capt., Vilber Lourmat SAS, France) (Ozkan et al., 2015).

### Cytotoxic evaluation

#### Cell culture materials

HeLa and PC3 cancer cell lines were obtained from Selcuk University, Faculty of Science, and Department of Biochemistry. Penicillin/Streptomycin, EMEM cell culture media, Ham's F-12 cell culture media, Fetal bovine serum (FBS), trypsin, MTT, ethanol, 2-prophanol, 60 × 15 mm corning plates and 75 cm^2^ corning flasks were purchased from Sigma-Aldrich (Sigma-Aldrich, USA).

#### Cell culture maintenance

Culture media were prepared with 10% fetal bovine serum (FBS) and 1% penicillin-streptomycin (Pen-Strep) solution. Prepared medium was kept at +4°C and warmed at 37°C before using. Cells (HeLa, PC3) were proliferated in incubator (37°C including 5% CO_2_). Stock cells were proliferated in 75 cm^2^ sterile corning flasks and experiment cell cultures were proliferated in 60 × 15 mm sterile petri dishes. In the logarithmic phase of the growth (when reached to ~80% cell confluency), cells were sub-cultured (Karakurt and Adali, 2016).

#### Preparation of plant extraction

Dilutions were made to obtain extract concentration of 10 mg/ml. The water extract was suspended in PBS and other extracts were suspended in DMSO (Dimethylsulfoxide)/water (1:5) and the diluted extracts were centrifuged at 10,000 × g during 2 min, and supernatants were filtrated (having the diameter of 0.22 μm pore size) and they were kept in −20°C.

#### MTT cell viability test

A total of 10.000 PC3 and HeLa cells were seeded in 96-well plates and allowed to fasten on the wells for 24 h. After 24 h, cells were treated with different doses of the tested extracts (0.1, 1, 2.5, 5, and 10 mg for water) (0.1, 1, 10, 100, and 1,000 μg for ethyl acetate and methanol) for 24 h. After exposure time completed, the medium was changed with EMEM and Ham's F12 medium supplemented with 0.5% FBS + 0.5 mg/ml MTT for HeLa and PC3 cancer cells, respectively. Then, they were incubated at 37°C with 5% CO_2_, for 4 h. After that time, the cells were treated with 3% SDS + 40 mM HCl/isopropanol for 15 min in order to dissolve the MTT-formazan crystals (Yerlikaya et al., 2016). The absorbance of each sample was recorded at 570 nm. Cell survival rate was calculated by using GraphPad Prism 3.03 software (GraphPad Software, Inc., La Jolla, CA, USA).

### Antimicrobial activity

Antimicrobial activity test was performed by disc diffusion method. Nutrient agar medium was used for test microorganisms and microorganisms were refreshed in 100 mm sterile petri dishes. Single colony of bacterial strain was inoculated in 20 ml nutrient broth medium for 24 h at 37°C incubator. After bacteria concentration was made visible in %9 sterile NaCI and its turbidity was standardized to 0.5 McFarland by adding bacterial suspension. Bacterial suspension seeded to 100 mm petri dishes. After the tested extracts were dissolved in DMSO and saturated on discs, they were incubated at room temperature for drying. They were placed on the inoculated petri plates with microorganisms for 24 h at 37°C incubator. Test was replicated at least three times and results were analyzed by measuring inhibition zone. Microorganisms were illustrated on Table 2.

**Table 2:** Microorganisms used for antimicrobial activity test.

***Microorganisms***	***Gram (+)***	***Gram (−)***
*Klebsiella pneumonia*		*X*
*Staphylococcus aureus ATCC 25923*	*X*	
*Staphylococcus hominis*	*X*	
*Proteus vulgaris*		*X*
*Escherichia coli*		*X*
*Serratia marcescens*		*X*
*Staphylococcus epidermidis*	*X*	
*Alfa streptococcus haemolyticua*	*X*	
*Enterococcus faecium*	*X*	
*Pseudomonas aeruginosa*		*X*
*Listeria monocytogenes ATCC 7644*	*X*	
*Enterococcus durans*	*X*	
*Salmonella kentucky*		*X*
*Enterobacter aerogenes ATCC 13048*		*X*

### Statistical analysis

All the assays were carried out in triplicate. The results were expressed as mean value and standard deviation (mean ± SD). Statistical differences between the extracts were analyzed by using one-way analysis of variance (ANOVA) followed by Tukey's honestly significant difference *post-hoc* test (α = 0.05). All the analysis was carried out using SPSS v22.0 software.

## Results and discussion

### Phytochemical composition

In the last decades, many studies highlighted biological activities of phenolic compounds, such as antioxidant, anticancer, antimicrobial, and anti-inflammatory. Flavonoids are the most abundant group of phenolics and considered as natural bioactive agents for designing novel functional products. In this sense, the amounts of total phenolics and flavonoids contents in *O. natrix* extracts were determined by Folin-Ciocalteu and AlCl_3_ methods. The greatest content of total phenolics was noticed in the ethyl acetate extract (60.19 mgGAE/g extract), followed by methanol (59.22 mgGAE/g extract) and water (35.12 mgGAE/g extract) extracts. Similar results were observed for flavonoid as well (Table 3). These findings were confirmed by several researchers who reported the higher concentration of total phenolics in ethyl acetate and methanol extracts (Do et al., 2014; Murugan and Parimelazhagan, 2014). In a previous study (Mhamdi et al., 2015), the total phenolic content of *O. natrix* in Tunisia was reported as 51 mgGAE/g, which was lower than that found in the present study. Moreover, some researchers were found to be different levels of total phenolics in several *Ononis* species, such as *O. spinosa* (3.09 mgGAE/g extract) (Orhan et al., 2012), *O. pubescens* (17.23 mgGAE/g extract), and *O. ornithopodioides* (20.96 mgGAE/g extract) (Sarikürkcü et al., 2016).

**Table 3:** Total phenolic and flavonoid contents, and free radical scavenging (DPPH and ABTS) activities of the extracts.

**Extracts**	**Total phenolic content (mgGAE/g extract)**	**Total flavonoid content (mgRE/g extract)**	**DPPH (mgTE/g extract)**	**ABTS (mgTE/g extract)**
Ethyl acetate	60.19 ± 1.67^*^	30.07 ± 0.39	56.60 ± 1.46	530.37 ± 0.40
Methanol	59.22 ± 0.09	28.40 ± 0.27	63.95 ± 1.45	521.80 ± 2.90
Water	35.12 ± 0.07	10.25 ± 0.23	63.38 ± 1.10	457.92 ± 2.29

*GAE, gallic acid equivalents; RE, rutin equivalents; TE, trolox equivalents*.

* *Values expressed are means ± S.D. of three parallel measurements*.

Individual phenolic constituents in the tested extracts were analyzed by HPLC-DAD and the results are given in Table 4. A total of 23 standard phenolics were used and 21 of them were identified in these extracts. Two compounds (rutin and hesperidin) were not detected in the extracts. Apigenin was quantified as the dominant phenolic in the ethyl acetate and methanol extracts. Other dominant phenolics were luteolin, epicatechin, and rosmarinic acid in these extracts. However, the most abundant phenolics in the water extract were benzoic acid and quercetin. In recent studies, the detected phenolics in *O. natrix* extracts possess a broad range of biological activities including antioxidant, anticancer, antimicrobial and enzyme inhibitory (López-Lázaro, 2009; Amoah et al., 2016; David et al., 2016). In this framework, the observed biological activities for *O. natrix* extracts might be explained with the presence of these phenolic components. To the best of our knowledge, this study is the first report which indicates comprehensive analysis of phenolic constituents of *O. natrix*. In this sense, the present findings could be opened new horizons for the genus *Ononis* and it' usages.

**Table 4:** Quantitative analysis for determination of phenolic components in the extracts (μg/g extract).

**No**.	**Phenolic compounds**	**Extracts**			**Analytical characteristics**			
		**Ethyl acetate**	**Methanol**	**Water**	**Linear range (ppm)**	***r*^2^**	**LOD (ppm)**	**LOQ (ppm)**
1	Gallic acid	8 ± 0.4	100 ± 0.8	58 ± 0.8	0.20–25.0	0.9993	0.075	0.227
2	Protocatechuic acid	10 ± 0.4	80 ± 0.6	234 ± 8	0.20–25.0	0.9991	0.086	0.260
3	(+)-Catechin	nd	152 ± 2	492 ± 20	0.90–113	0.9988	0.172	0.522
4	*p*-Hydroxybenzoic acid	44 ± 4	106 ± 4	200 ± 4	0.20–25.0	0.9994	0.007	0.020
5	Chlorogenic acid	210 ± 2	nd	166 ± 2	0.35–45.0	0.9988	0.080	0.241
6	Caffeic acid	42 ± 4	34 ± 4	90 ± 4	0.16–21.0	0.9993	0.054	0.162
7	(-)-Epicatechin	310 ± 16	950 ± 24	nd	0.50–66.0	0.9990	0.170	0.514
8	Syringic acid	22 ± 0.2	66 ± 4	86 ± 4	0.05–12.0	0.9995	0.030	0.090
9	Vanillin	2 ± 0.1	10 ± 0.2	nd	0.08–10.0	0.9995	0.020	0.060
10	*p*-coumaric acid	8 ± 0.6	34 ± 2	64 ± 2	0.04–6.0	0.9996	0.066	0.199
11	Ferulic acid	8 ± 0.4	90 ± 0.4	130 ± 6	0.12–17.0	0.9993	0.004	0.011
12	Sinapic acid	nd	nd	54 ± 2	0.12–17.0	0.9993	0.017	0.053
13	Benzoic acid	50 ± 0.6	50 ± 0.6	1386 ± 26	0.85–55.0	0.9998	0.111	0.335
14	*o*-Coumaric acid	82 ± 2	82 ± 2	nd	0.24–32.0	0.9988	0.023	0.069
15	Rutin	nd	nd	nd	0.40–56.0	0.9989	1.113	3.373
16	Hesperidin	nd	nd	nd	0.43–55.0	0.9992	1.080	3.280
17	Rosmarinic acid	76 ± 2	1960 ± 22	nd	0.02–7.0	0.9998	0.148	0.447
18	Eriodictyol	nd	nd	116 ± 4	0.33–21.0	0.9998	0.140	0.410
19	*trans*-Cinnamic acid	18 ± 1	66 ± 1.2	10 ± 1	0.02–7.0	0.9998	0.148	0.447
20	Quercetin	nd	nd	1356 ± 42	0.40–55.0	0.9999	0.013	0.040
21	Luteolin	756 ± 56	908 ± 56	1006 ± 56	0.13–17.0	0.9999	0.020	0.060
22	Kaempferol	nd	288 ± 6	222 ± 4	0.05–15.0	0.9996	0.021	0.062
23	Apigenin	4136 ± 94	3314 ± 86	850 ± 24	0.17–11.0	0.9997	0.034	0.104

*nd, not detected*.

### Antioxidant capacity

In order to evaluate antioxidant ability of *O. natrix* extracts, several methods were performed: free radical scavenging (DPPH and ABTS), reducing power (CUPRAC and FRAP), phosphomolybdenum and metal chelating assays. DPPH and ABTS assays are widely utilized to examine radical scavenging activities of plant extracts or synthetics. These assays are based on the reduction of ABTS^+^ and DPPH^·^ in the presence of antioxidants and the changes (from blue to white in ABTS; from purple to yellow in DPPH) can spectrophotometrically be recorded. In addition, the assays reflect to hydrogen donating abilities of antioxidants. As shown in Table 3, the methanol and water extracts had similar DPPH scavenging abilities whereas the lowest ability was observed in the ethyl acetate extract. However, ABTS radical scavenging abilities can be ranked as ethyl acetate> methanol>water. The apparent differences can be attributed to nature of these radicals (ABTS assay is considered as both hydrophilic and lipophilic antioxidant systems, while DPPH is only hydrophobic systems). Our findings are consistent with previous results, which were reported different results for DPPH and ABTS (Kim et al., 2002; Zengin et al., 2015, 2017; Bouhlali et al., 2017).

Reducing power is an important way in the antioxidant mechanisms and it reflects electron-donating abilities of antioxidants. From this point, CUPRAC and FRAP assays were performed to detect reducing abilities of *O. natrix* extracts (Table 5). By using CUPRAC assay (from Cu^2+^ to Cu), the ethyl acetate extract showed the strongest reducing ability, followed by methanol and water extracts. These results are in line with the total phenolic and flavonoid contents. Nevertheless, the FRAP (from Fe^3+^ to Fe^2+^) abilities decreased in that order water (106.12 mgTE/g extract)>ethyl acetate (90.71 mgTE/g extract)>methanol (79.73 mgTE/g extract). This finding for water extract in FRAP assay might be related to complex interactions of phytochemical (antagonistic etc.) described as Peña-Cerda et al. (2017) and (Hmid et al., 2017).

**Table 5:** Total antioxidant capacity (phosphomolybdenum assay), reducing power (CUPRAC and FRAP assays) and metal chelating activities of the extracts.

**Extracts**	**Phosphomolybdenum (mmolTE/g extract)**	**CUPRAC (mgTE/g extract)**	**FRAP (mgTE/g extract)**	**Metal chelating (mgEDTAE/g extract)**
Ethyl acetate	1.27 ± 0.07^*^	183.37 ± 0.02	90.71 ± 1.79	9.16 ± 0.17
Methanol	1.53 ± 0.08	170.51 ± 0.57	79.73 ± 1.17	12.02 ± 1.40
Water	1.13 ± 0.03	94.16 ± 0.99	106.12 ± 0.44	14.49 ± 0.64

*TE, trolox equivalents; EDTAE, EDTA equivalents*.

* *Values expressed are means ± S.D. of three parallel measurements*.

Phosphomolybdenum assays is proposed by Prieto et al. (1999), which is based on the reduction of Mo (VI) to Mo (V) by the antioxidants and then a green phosphate/Mo (V) complex occurs at the acidic pH. The assay is also considered as one of total antioxidant capacity assays. Within *O. natrix* extracts, the methanol extract (1.53 mmolTE/g extract) exerted the strongest ability in phosphomolybdenum assay, followed by ethyl acetate (1.27 mmolTE/g extract) and water (1.13 mmolTE/g extract) extracts (Table 4). In the literature, several researchers reported different approaches for a correlation between total phenolic and the reduction of Mo (VI) to Mo (V) (Albayrak et al., 2010; Kocak et al., 2016; Amessis-Ouchemoukh et al., 2017). At this point, this activity for the methanol extract may be related to the presence of other antioxidants as well as phenolics.

Transition metal ions, such as iron and copper are involved in the production of hydroxyl radical via Fenton and Haber-Weiss reactions. In this sense, the metal chelating ability is considered as an important way in the antioxidant mechanisms. Contrary to expectations, the metal chelating ability significantly differs from other antioxidant assays and the strongest activity was observed in water extract with 14.49 mgEDTAE/g, while the ethyl acetate extract had the lowest ability (Table 5). These findings are consistent with previous studies, which reported no correlation between total phenolic and metal chelating ability (Silva et al., 2011; Hatami et al., 2014; Khorasani Esmaeili et al., 2015). In fact, the metal chelating abilities of phenolics were described as a minor way in theirs antioxidant properties by Rice-Evans et al. (1996).

### Enzyme inhibitory effect

Alzheimer's diseases (AD) and diabetes mellitus (DM) are considered as major public health problems and their incidence tends to increase at an alarming rate (more than 35.6 million AD patients in worldwide and is expected to be more than 100 million AD patients by 2050). The development of new therapeutic approaches (also in terms of non-pharmacological therapy supplement) for these diseases has become a pressing issue. Among the therapeutic approaches, the inhibition of key enzymes is considered as one of most effective strategy (Wimo and Prince, 2010). Cholinesterase inhibitors are currently used as a drug for the treatment of AD. The inhibitors increase brain acetylcholine (ACh) levels by preventing the breakdown of ACh and this case is very important to cognitive function (Menichini et al., 2014). Again, α-amylase and α-glucosidase release glucose from larger carbohydrates (especially starch) and are key enzymes in DM glucose level control (Kubinova et al., 2014). In this direction, both α-amylase and α-glucosidase inhibitors are promising drugs in the treatment of DM. In addition, tyrosinase is a main enzyme in the synthesis of melanin and thus tyrosinase inhibitors are useful for controlling hyperpigmentation problems. Within the framework of these information's, many enzyme inhibitors are synthetically produced. However, most of them show limited effectiveness especially related to side effects, such as gastrointestinal disturbances, and toxic properties (Kumar et al., 2011). Therefore, many scientists have focused on naturally occurring compounds from plants as potential sources of either new or safe effective inhibitors.

Thus, we tested enzyme inhibitory effects of *Ononis* extracts against to cholinesterases, tyrosinase, amylase and glucosidase. The results are represented in Table 6. The ethyl acetate extract exhibited the strongest cholinesterase inhibitory effect on both AChE (1.46 mgGALAE/g extract) and BChE (0.93 mgGALAE/g extract). However, the water extract was not active on BChE. The observed activity for ethyl acetate extract may be linked to higher level of phenolics in the extracts. Our findings were confirmed by several researchers (Kennedy and Wightman, 2011; Roseiro et al., 2012; Mazlan et al., 2013; Hlila et al., 2015), who reported a linear correlation between phenolic content and anti-cholinesterase abilities. Interestingly, the ethyl acetate extract did not have any inhibitory effect on tyrosinase and the water extract had remarkable anti-tyrosinase effect with 52.81 mgKAE/g extract. As for amylase and glucosidase inhibition, the ethyl acetate (0.74 mmolACAE/g and 17.52 mmolACAE/g) and methanol extracts (0.59 mmolACAE/g and 19.94 mmolACAE/g) had the greatest abilities compared to water extract. The higher concentrations of phenolics in these extracts may be responsible for their anti-diabetic effects. In accordance with our findings, some phenolic components were reported as anti-diabetic agents (Tundis et al., 2010; Etxeberria et al., 2012; Ríos et al., 2015). From this point, molecular approaches could be valuable to understand interactions of enzymes and phenolics. To the best of our knowledge, this study is the first report on *Ononis natrix* subsp. *hispanica*. Taken together, this study could provide a starting point on this species and could open new perspectives for designing novel functional products.

**Table 6:** Enzyme inhibitory effects of the extracts.

**Extracts**	**AChE ınhibition (mgGALAE/g extract)**	**BChE ınhibition (mgGALAE/g extract)**	**Tyrosinase ınhibition (mgKAE/g extract)**	**α-amylase ınhibition (mmolACAE/g extract)**	**α-glucosidase ınhibition (mmolACAE/g extract)**
Ethyl acetate	1.46 ± 0.07^*^	0.93 ± 0.01	na	0.74 ± 0.02	17.52 ± 0.28
Methanol	1.29 ± 0.05	0.64 ± 0.06	11.20 ± 2.30	0.59 ± 0.01	19.94 ± 0.11
Water	0.02 ± 0.01	na	52.81 ± 5.15	0.17 ± 0.01	3.69 ± 0.17

*GALAE, galanthamine equivalents; ACAE, acarbose equivalents; KAE, kojic acid equivalents; na, not active*.

* *Values expressed are means ± S.D. of three parallel measurements*.

### Molecular docking

**Figure 2:**
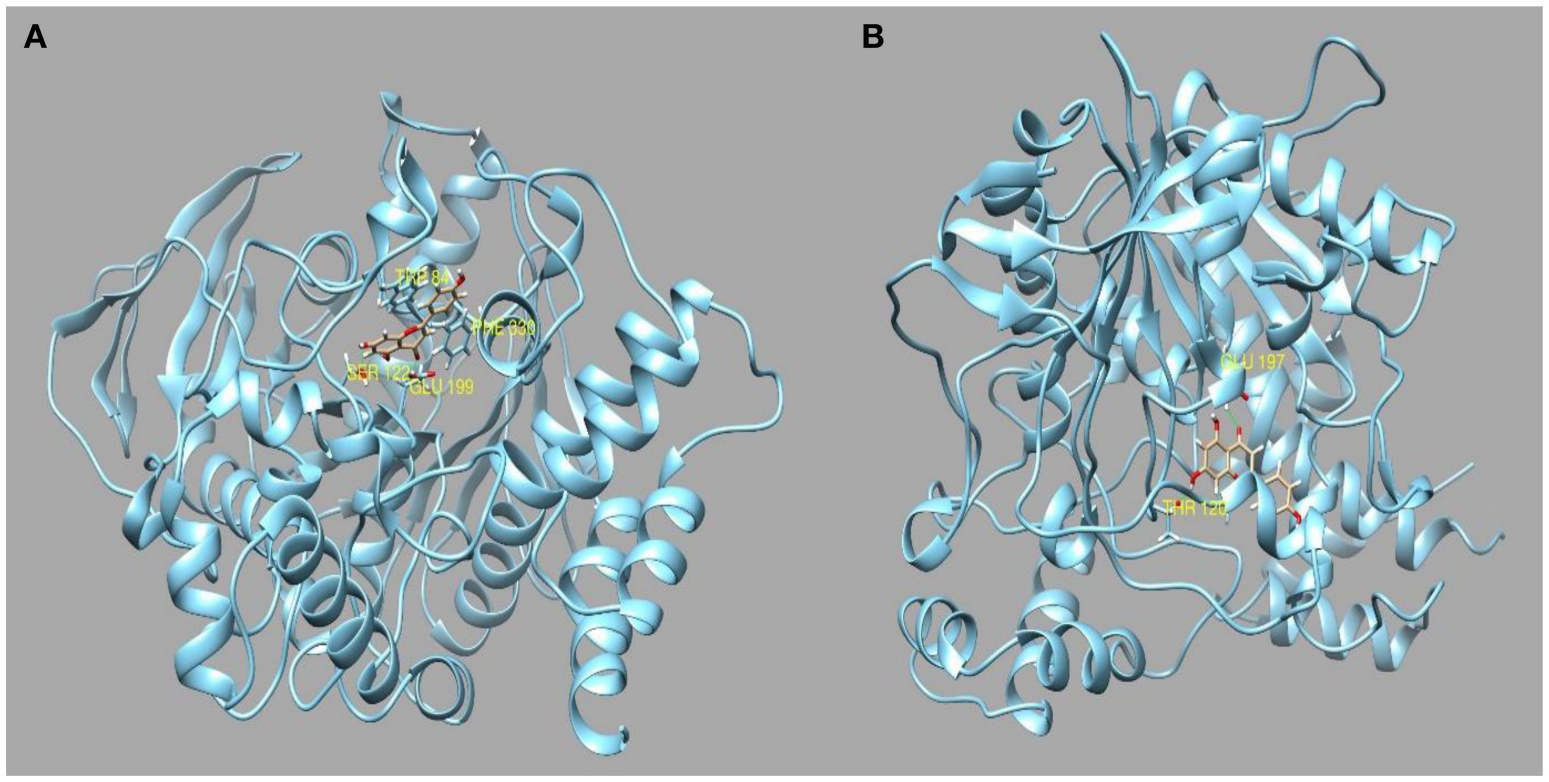
**(A)** Best pose of apigenin docked to AChE and **(B)** to BChE.

**Figure 3:**
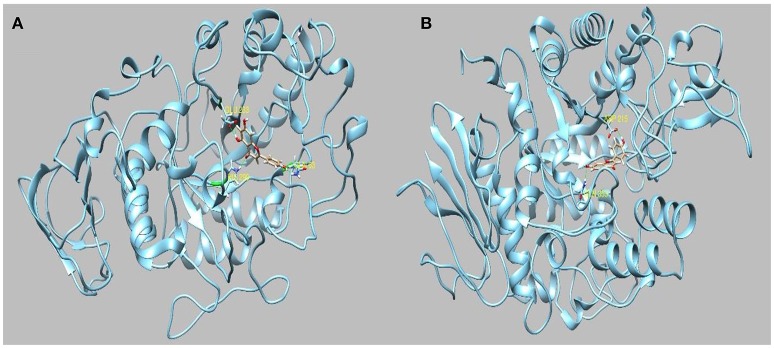
**(A)** Best pose of apigenin docked to α-amylase and **(B)** to α-glucosidase.

**Figure 4:**
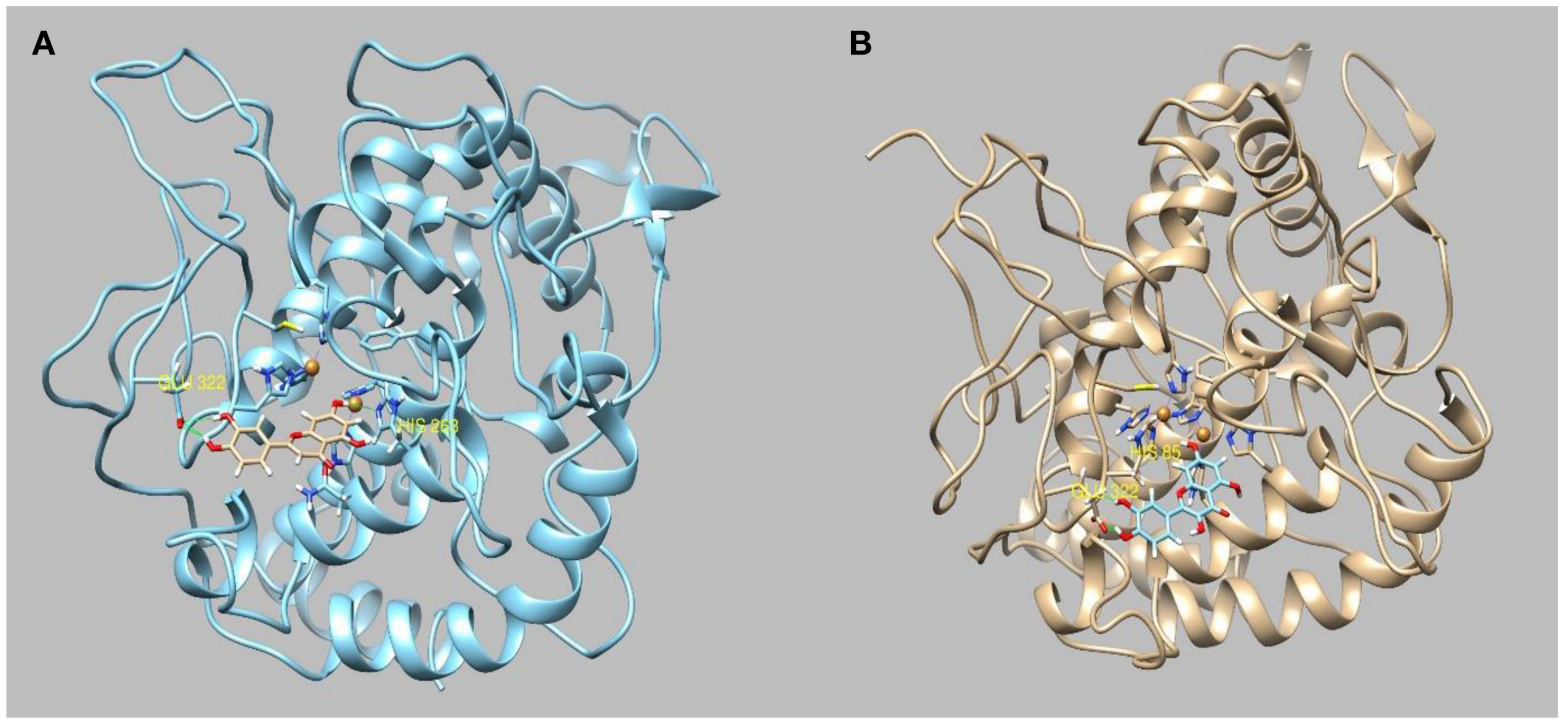
Best pose of **(A)** luteolin and **(B)** quercetin docked to tyrosinase.

Evidence from the literature supports the inhibitory action of apigenin on AChE and BChE (Katalinić et al., 2010; Xie et al., 2014). Apigenin was also reported to induce moderate inhibitory effect on α-amylase (Li et al., 2014; Wulan et al., 2014) and on α-glucosidase. On the other hand, we have found that the water extract possesses good inhibition activity toward tyrosinase. This may be attributed to the presence of luteolin and quercetin. In light of the observed inhibitory activity, *in silico* molecular docking simulation was used to investigate the interactions between apigenin to AChE, BChE, amylase and glucosidase, whereas luteolin and quercetin have been selected as principal inhibitors of tyrosinase. The most representative enzyme-ligand complexes were reported in Figures 2–4.

Apigenin has been previously reported to inhibit both AChE and BChE by IC50 of 7.72 micro/Mol (Balkis et al., 2015) and 37.4 micro/Mol (Katalinić et al., 2010), respectively. The best docked pose interacts with AChE in the binding pocked by establishing two hydrogen bonds to Glu199 and Ser122 and two pi-pi stacks to Phe330 and Trp 84 with a docking score of −9.607 and a binding energy of −60.64 Kcal/Mol. Apigenin also interacts with several residues present in the binding pocket of BChE forming two hydrogen bonds to Thr120 and Glu197 with a docking score of −9.459 and a binding energy of −52.54 kcal/Mol. Also, considering the abundance in the ethyl acetate and methanol extracts, apigenin could be also responsible for the amylase and glucosidase inhibiting activity. Indeed, it has been previously reported that apigenin was an efficacious inhibitor of α-glucosidase with a IC50 of and a moderate α-amylase inhibitor with a IC50 of 10.5 μ/Mol (Zeng et al., 2016).

Apigenin interacts with α-glucosidase by forming two hydrogen bonds to Asp215 and Gln353 and with the enzymatic cavity of α-amylase by forming three hydrogen bonds to Gln63, His299, and Glu233. The aqueous extract has shown a strong activity toward tyrosinase. The most abundant substances present in the extracts are benzoic acid, luteolin and quercetin. All of these substances have been demonstrated to have an tyrosinase inhibitory activity in *in vitro* experiments (IC50 = quercetin 0.13 mM) (Chen and Kubo, 2002). However, benzoic acid reported a scarce docking score (−5.1) and a positive binding energy (+41.7 Kcal/Mol) which indicate none or very little affinity for the binding pocket of the enzyme, whereas the luteolin and quercetin have obtained the best binding energy. (−28 Kcal/Mol and −29.31 Kcal/Mol, respectively).

However, it is noteworthy to highlight that the plant extracts contain several phenolic compounds which in less extent are able to inhibit the tested enzymes all with non-competitive mechanism as demonstrated in our previous paper (Picot et al., 2017). Inhibitory action of the plant extracts on AChE, BChE, tyrosinase, α-amylase, and α-glucosidase might be due to the concerted action of several phenolic compounds rather than a single molecule.

### DNA protective effects

**Figure 5:**
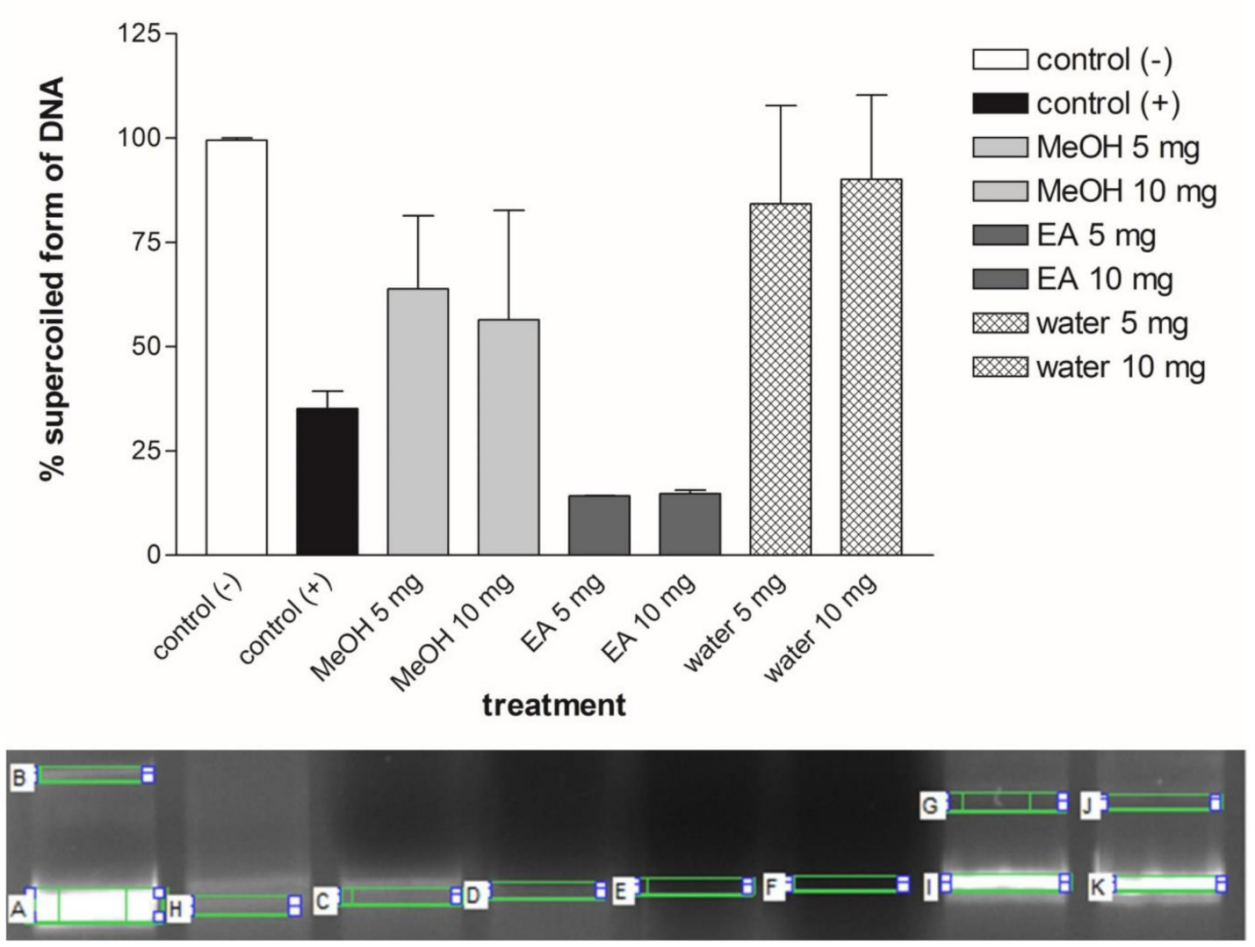
percentage of DNA protection analysis and gel view of plasmid DNA protection test for each *Ononis natrix* extracts. A,B: (+) control, H: (−) control, C: 5 mg methanol extract, D: 10 mg methanol extract, E: 5 mg ethyl acetate extract, F: 10 mg ethyl acetate extract, I-G: 5 mg water extract, K-J: 10 mg water extract. Striped bars indicate significantly different applications.

Various stress factors, such as oxidative stress, acid, alkaline, UV, and heavy metals can damage DNA. ROS formed by hydroxyl radicals cause DNA strand breakage that is brought about carcinogenesis, mutagenesis and cytotoxicity (Golla and Bhimathati, 2014). Here, we targeted to measure the DNA damage by Fenton reaction-mediated oxidative stress. Fenton's reagent (Fe^2+^ + H_2_O_2_ → Fe^3+^ + OH^−^ + ⋅OH) produces the highly deleterious hydroxyl radicals that damage the cellular components, such as DNA, lipid and proteins. The scavenging effect of extract was tested in plasmid nicking assay. Figure 5 illustrates the DNA protection activity of the *Ononis* extracts with two different concentrations (5 and 10 mg/ml). *Ononis* extracted in water (10 mg/ml) had the most effective on protection of DNA with 78%, followed by water (5 mg/ml) (70%) and methanol extract with 5 mg/ml (53%). Except for the ethyl acetate extract (5 and 10 mg/ml), water and methanol extracts protected supercoiled form of pDNA and indicated DNA protection activity.

In the present study, the water extract had the most protection activity on DNA. This can be arisen from availability of different metabolites in water extract. Quercetin and sinapic acid are only metabolites which found in this extract. So, it can be concluded that these chemicals may be responsible for protection of DNA from Fenton reagent. Like water extract, the methanol extract also contained specific metabolite, such as rosmarinic acid. In this sense, the observed DNA protection ability for the methanol extract can be arisen from presence of higher amount of rosmarinic acid. Therefore, it seems that different ingredients of the extracts may cause formation of DNA protection property for the tested extracts. According to DNA protection assay results, water extract of *Ononis natrix* showed the most protective activity for DNA. This result may be explained by determination of phenolic components in the extracts using HPLC analysis. As indicated in Table 4, rosmarinic acid and quercetin were the most dissolved components in methanol and water extracts of *Ononis natrix*, respectively. So, it can be concluded that rosmarinic acid and quercetin can be considered as potential chemicals for protection of DNA from free radicals, hydroxyl radicals and mutagens. Quercetin is a well-investigated antioxidant known as promising molecule for pharmacological studies. In a comprehensive research for DNA protection ability of quercetin, its supplementation indicated protection of nuclear DNA from oxidative damage and significant genoprotective activity on mitochondrial DNA (Potenza et al., 2008). In a different study, three polyphenolic compounds including luteolin, quercetin and rosmarinic acid, were investigated for their protective effects against oxidative DNA damage (Silva et al., 2008). They found that all of these phenolics were protection agents against oxidative stress-induced DNA damage. In another search, protective activity of pure rosmarinic acid and elagic acid were evaluated. It has been found that they have equal effect on declining AAPH-induced oxidative DNA damage (Vattem et al., 2006). These results are in accord with our study indicating that methanol and water extracts of *Ononis natrix* can be considered as valuable DNA protectors because of presence of rosmarinic acid and quercetin.

### Cytotoxic effects

**Figure 6:**
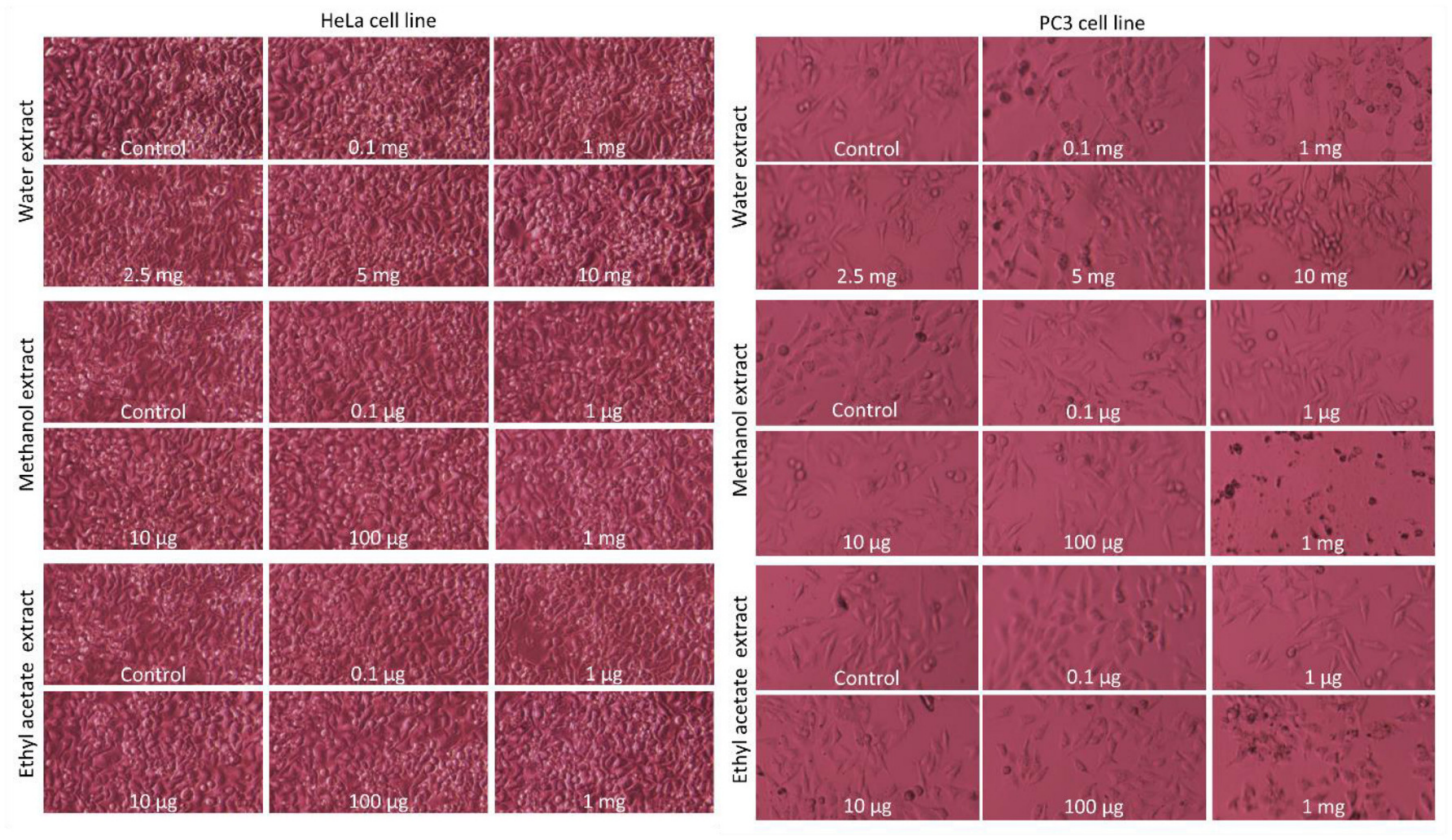
Cells morphology of HeLA and PC3 cancer cell lines after application of different extracts of *Ononis natrix*. Control and different concentrations of extracts were tested in HeLA or PC3 cancer cell lines and indicated for each figures.

**Figure 7:**
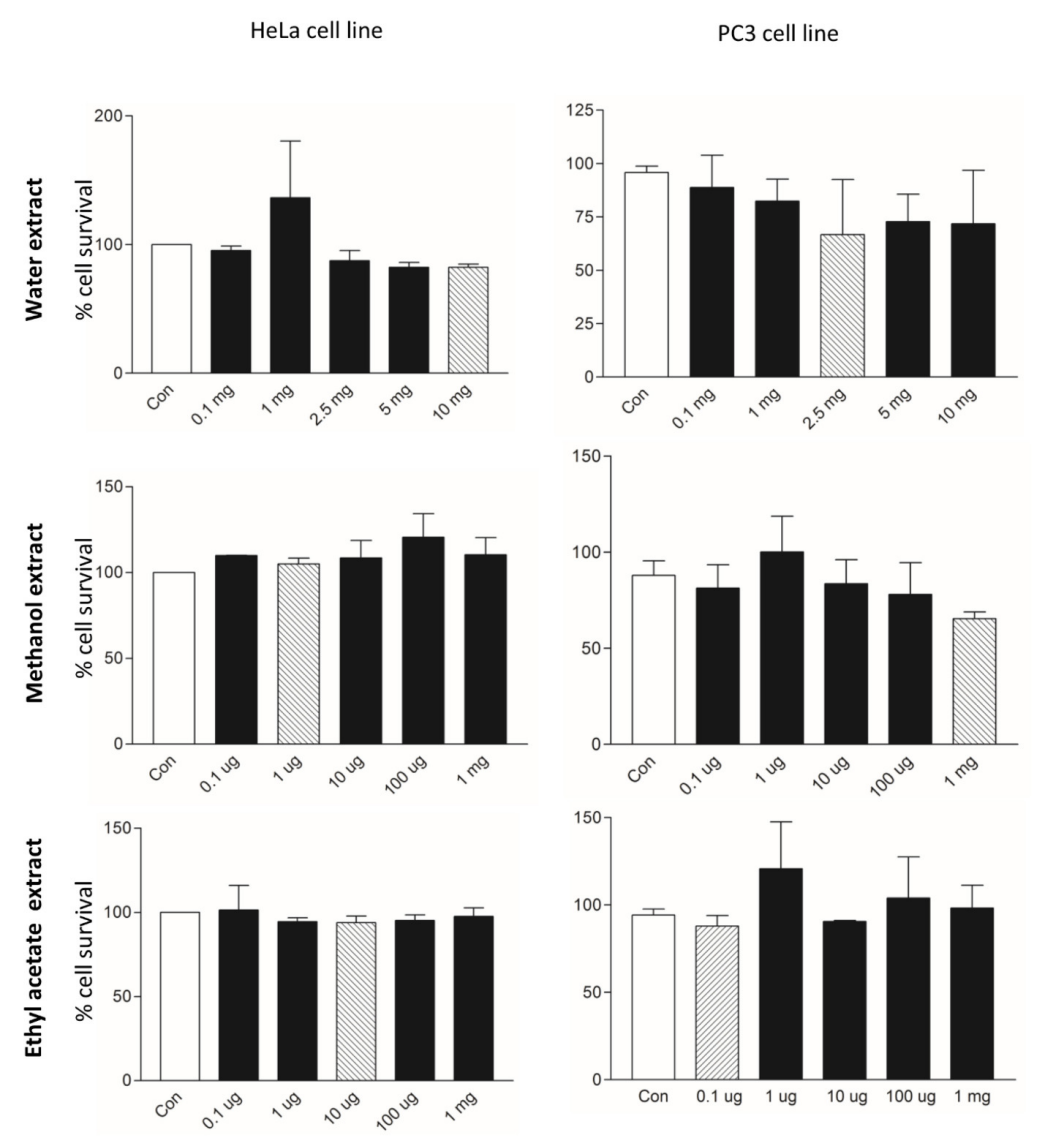
Cell viability assay (MTT) analysis of HeLA and PC3 cancer cell lines after application of different extracts of *Ononis natrix*. White bars and striped bars indicate controls and significantly different applications, respectively.

The cytotoxic effects of *Ononis natrix* on the survival of HeLa and PC3 cells were determined by MTT cell viability assay. HeLa cervical cancer cell line and PC3 prostate cancer cell line were treated with these extracts with different concentrations for 24 h. Cells morphology was observed under inverted microscope and illustrated in Figure 6. According to morphological appearance, there was no significant apoptotic effect on *Ononis natrix* extracts for HeLa cell line. However, the water and methanol extracts caused initiation of apoptosis for PC3 cell line. To indicate effect of three extracts on cancer cell lines, cell viability assay (MTT) was also conducted. Although there was a cytotoxic activity for HeLa cells after application of the water extract, no significant decline was observed in cancer cell numbers compared to control (Figure 7). On the other hand, 2.5 mg water extract, 1 mg methanol extract and 0.1 μg ethyl acetate extract caused a significant cell number reduction on PC3 cancer line (Figure 7). Ethyl acetate extract indicated cytotoxic activity with the smallest concertation (0.1 μg) among all extracts. 2.5 mg of the water extract showed dose-independent cytotoxic effect on PC3 cell line since there was no effect for 5 mg and 10 mg water extracts. It has been considered that concentration of water extract more than 2.5 mg may be toxic against healthy or normal viable cells. Thus, it can be concluded that 2.5 mg is a moderate concentration for cytotoxicity on PC3 cancer cell line. It was also monitored that other treatments had cytotoxic effect whereas, they were not significantly different from control sample anymore.

According to reports, about 60% of anticancer drugs are isolated from plant-derived natural compounds. These medicinal plants highly contain polyphenolic compounds which are actually inhibitors for cancer development (Sukumaran et al., 2016). Cytotoxic and apoptotic effects of the studied extracts on HeLa and PC3 cell lines were investigated. Although there was no significant variation on HeLa cells, 2.5 mg of water and 1 mg methanol extracts leaded to cytotoxic activity compared to control in PC3. Some of phenolic compounds show anti-proliferative effect which depends on polarity of compounds. Cytotoxic effect of methanol extract can be explained with the presence of rosmarinic acid, apigenin, and epicatechin. Effect of anti-carcinogenic and scavenging of reactive oxygen radicals of rosmaniric acid were particularly searched in *Perilla frutescens* (Osakabe et al., 2004). The body's response to cancer showed parallel effects with the inflammatory response. Since, inflammatory cytokine networks may affect survival, growth, mutation, proliferation, differentiation and relocation of tumor and stromal cells (David, 1988; Balkwill and Mantovani, 2001; Osakabe et al., 2004). Rosmaniric acid is a water-soluble polyphenolic compound and has function on inhibition of angiogenesis (Huang and Zheng, 2006). There was a potential evidence that ROS acts as initiating angiogenesis and onset of cancer mechanism. It was proved that hydrogen peroxide stimulated angiogenesis *in vitro* condition (Shono et al., 1996; Yasuda et al., 1999; Huang and Zheng, 2006). Rosmaniric acid reduced ROS expression, hydrogen peroxide level and VEGF (Vascular Endothelial Growth Factor) level that are important on cancer development (Huang and Zheng, 2006). Furthermore, the previous report which indicated effect of methanolic extract of plant species on antioxidant activity and examination of level of rosmaniric acid (Tepe, 2008). In addition, in our previous study, three extracts (ethyl acetate, methanol and water) of two *Potentilla* species (*Potentilla reptans and P. speciosa*) showed cytotoxic activity against A549 andMCF-7 due to some phenolic compounds (Uysal et al., 2017). The findings of this analysis suggest that one or more than one phenolic compounds and their interaction may cause initiation of apoptosis in cancer cell lines. However, further investigation should be performed in *in vivo* test to arrive at a definite judgment for inhibition of cancer development.

### Antimicrobial effects

**Figure 8:**
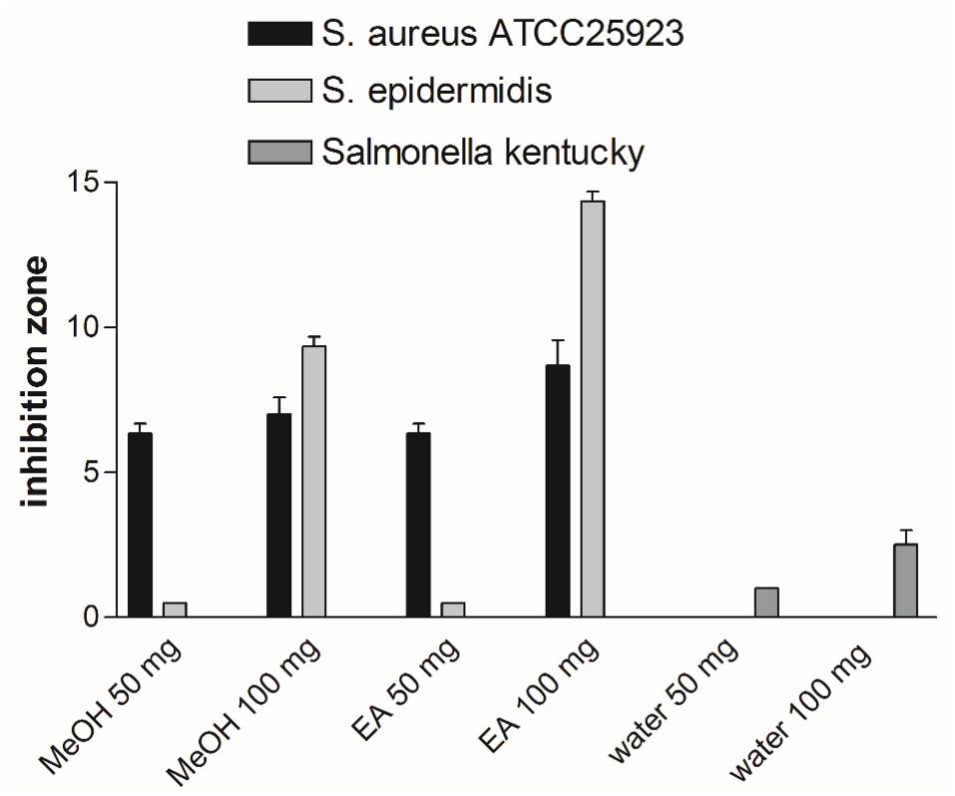
Antimicrobial activity of methanol, ethyl acetate and water extracts of *Ononis natrix* against to *S. aureus ATCC 25923, S. epidermidis* and *S. kentucky*.

Antimicrobial activity of the extracts from *O. natrix* subsp. *hispanica* were searched against to 13 bacteria and 1 fungus by the disk diffusion method. The extracts were loaded the disks at the concentration of 10, 50, and 100 mg. According to results, 50 and 100 mg of methanol and ethyl acetate extracts showed the best antibacterial effect against to *Staphylococcus aureus ATCC 25923* and *Staphylococcus epidermidis* strains (Figure 8). In addition, 100 mg water extracts had a small effect on *Salmonella kentucky*. It was realized that extracts possessed more protective effect against to gram positive bacteria strains. In addition, concentration-dependent effect was also observed. For example, *S. epidermis* stain was more sensitive against to 100 mg of methanol and ethyl acetate extracts than 50 mg ones. Methanol and ethyl acetate extracts of *O. natrix* subsp. *hispanica* were more effective than water extract for antimicrobial analysis. The results of this analysis support the idea that because of higher total phenolic and flavonoid contents of methanol and ethyl acetate extracts, they indicated more antimicrobial activity than water one.

*Staphylococcus aureus* (ATCC 25923) is a gram positive bacterium and causes several human infections and leads to sanitary problems. *S. aureus* also shows resistance to some antibiotics and drugs particularly to methicillin (Hannan et al., 2008). Resistance problems induced to researchers to tend for seeking novel therapy. *S. epidermidis* together with *S. aureus* lead to severe infectious diseases (Otto, 2009). *S. epidermidis* is more being tend to cause infectious diseases due to developing persistent infection on human skin (Otto, 2009). Researchers are seeking the mechanisms by which *S. epidermidis* promotes diseases (Otto, 2009). In a study of Sayari et al. (2016) *Ononis natrix* leaves extracts were tested against to 9 bacterial strains (5 gram-negative and 4 gram-positive). Consistently with our study, *S. aureus* were the most susceptible against to *Ononis natrix* leaves extracts. In another study, *S. aureus* (ATCC 25923), and *E. coli* (ATCC 25922) were found to have more sensitivity against the *Ononis natrix* essential oils with inhibition zone as 27 and 25, respectively (Elamrani and Benaissa, 2010).

## Conclusion

In recent years, pharmaceuticals and functional ingredients from plant sources have been of paramount interest. In this sense, chemical profile and biological abilities of *O. natrix* subsp. *hispanica* were investigated in the current work. As far as we aware, this work is the first report on this species. The tested extracts, especially ethyl acetate and methanol, exhibited notable biological effects correlated with higher levels of bioactive compounds. The results obtained from the present work provide a new framework for utilization of the genus *Ononis* and as a result, *O. natrix* subp. *hispanica* could be suggested as a natural source of bioactive agents, such as antioxidant, antimicrobial and anticancer. We hope that our research will serve a base for future studies for *in vivo* and bioavailability studies on *O. natrix* subsp. *hispanica*.

## Author contributions

SY, GZ, MB, and YC set up and carried out experiments. AM and AA executed data analysis.

### Conflict of interest statement

The authors declare that the research was conducted in the absence of any commercial or financial relationships that could be construed as a potential conflict of interest.
